# A cricket Gene Index: a genomic resource for studying neurobiology, speciation, and molecular evolution

**DOI:** 10.1186/1471-2164-8-109

**Published:** 2007-04-25

**Authors:** Patrick D Danley, Sean P Mullen, Fenglong Liu, Vishvanath Nene, John Quackenbush, Kerry L Shaw

**Affiliations:** 1 Department of Biology, University of Maryland, College Park, MD 20742, USA; 2 Department of Biostatistics and Computational Biology, Dana-Farber Cancer Institute, Boston, MA 02115, USA; 3 The Institute for Genomic Research, 9712 Medical Center Drive, Rockville, MD 20850, USA; 4 Department of Cancer Biology, Dana-Farber Cancer Institute, Boston, MA 02115, USA; 5 Department of Biostatistics, Harvard School of Public Health, Boston, MA 02115, USA

## Abstract

**Background:**

As the developmental costs of genomic tools decline, genomic approaches to non-model systems are becoming more feasible. Many of these systems may lack advanced genetic tools but are extremely valuable models in other biological fields. Here we report the development of expressed sequence tags (EST's) in an orthopteroid insect, a model for the study of neurobiology, speciation, and evolution.

**Results:**

We report the sequencing of 14,502 EST's from clones derived from a nerve cord cDNA library, and the subsequent construction of a Gene Index from these sequences, from the Hawaiian trigonidiine cricket *Laupala kohalensis*. The Gene Index contains 8607 unique sequences comprised of 2575 tentative consensus (TC) sequences and 6032 singletons. For each of the unique sequences, an attempt was made to assign a provisional annotation and to categorize its function using a Gene Ontology-based classification through a sequence-based comparison to known proteins. In addition, a set of unique 70 base pair oligomers that can be used for DNA microarrays was developed. All Gene Index information is posted at the DFCI Gene Indices web page

**Conclusion:**

Orthopterans are models used to understand the neurophysiological basis of complex motor patterns such as flight and stridulation. The sequences presented in the cricket Gene Index will provide neurophysiologists with many genetic tools that have been largely absent in this field. The cricket Gene Index is one of only two gene indices to be developed in an evolutionary model system. Species within the genus *Laupala *have speciated recently, rapidly, and extensively. Therefore, the genes identified in the cricket Gene Index can be used to study the genomics of speciation. Furthermore, this gene index represents a significant EST resources for basal insects. As such, this resource is a valuable comparative tool for the understanding of invertebrate molecular evolution. The sequences presented here will provide much needed genomic resources for three distinct but overlapping fields of inquiry: neurobiology, speciation, and molecular evolution.

## Background

Identifying the genetic basis of interesting phenotypic variation in non-model systems is often limited by the lack of sophisticated molecular resources, such as complete genome sequences and DNA microarrys, that are available in model genetic taxa such as *Drosophila *[1], *Anopheles *[2], *Caenorhabditis *[3] and *Apis *[4]. However, the declining costs of developing genomic tools and the proliferation of accessible methods by which these tools can be generated holds promise for genomic-scale studies in organisms that offer profound insights into fundamental biological questions. Thus, there is a growing need to develop better genomic resources for these emerging systems.

The Orthoptera contain many such emerging systems. Consisting of over 25,000 species [5], the order Orthoptera is composed of two major lineages, the crickets and katydids (Ensifera) and the grasshoppers (Caelifera) [6,7] which diverged approximately 300 MYA. While well known for their economic impact on world-wide agriculture [8-13], they have been intensively studied in a wide variety of biological areas. For example, orthopterans have been used to study various aspects of neurobiology [14-17], physiology [18-21], behavior [10,22-24], development [17,25-28], sexual selection [29-35], and evolution [7,32,36-43]. However, very few genomic tools have been developed for this group of insects.

While genomic studies of many orthoptera are ongoing [44,45], large scale genomic resources have been developed for only one species in this order, *Locusta migratoria *(Caelifera) [45,46]. Research on *Locusta *has produced 12,161 unique sequences and provides a necessary counterpoint to the heavy phylogenetic bias in extant genomic resources. [47-50]. However, as described above, orthopterans are a phylogenetically diverse lineage which are being used to study a broad set of biological questions. The Gene Index presented here was developed to address three distinct but overlapping areas of orthopteran biology: neurobiology, speciation, and evolution.

For over 50 years, the Orthoptera have been used as a neurobiological model system by which the relationship between neural activity, muscular response and behavior are studied [51]. In particular, the study of orthopteran flight and song, or stridulation, have provided valuable insights into the physiological basis of behavior and the structure and function of Central Pattern Generating (CPG) circuits [52-55]. CPG circuits are responsible not only for orthopteran flight and song, but also for nearly all vital functions, such as circulation, respiration, digestion and locomotion, in both vertebrates and invertebrates. Since at least 1973, neuroethologists have called for the development of genetic tools to understand the creation, function, and diversification of the neural circuits responsible for cricket stridulation [56]. One result has been the analysis of the inheritance of species-specific songs [57,58] and a quantitative trait locus study of song (Shaw et al. in press). Yet the tools necessary to study the action and influence of individual genes remain largely absent. The EST's of this Gene Index, since they are derived from a nerve cord library, contain genes expressed in nervous system. Many of the EST's identified here may be involved in the construction of the flight and/or stridulation CPG.

Furthermore, our study organism, *Laupala kohalensis*, is a superb organism with which to investigate the genetic basis of CPG construction and evolution. The 38 species of *Laupala *have diverged within the past five million years [59]. The diversification of *Laupala *has been extraordinarily rapid, as *Laupala *contains the fastest diversifying arthropod clade recorded to date [59]. The radiation is also noteworthy for the extremely limited number of features that distinguish species. Members of this genus appear morphologically and ecologically similar and many closely related species often differ by fewer than 0.1% of nuclear gene bases [60]. However, pulse rates of male calling songs have diverged extensively in *Laupala *[61]. Given the diversity of pulse rate CPG's in this clade and the limited amount of genetic divergence that separates species, the release of the *Laupala *Gene Index will provide an extraordinary genomic tool by which CPG evolution may be studied.

In addition to providing a powerful platform for comparative studies of CPG evolution, *Laupala *is a well-developed model system for the study of reproductive isolation and the formation of species [33,34,38,59,60,62-66]. The 38 species within this genus are believed to have diverged in part via coordinated evolution in male song and female acoustic preference [33,34,65]. While there exists an extensive body of literature on the evolution of sexual isolation and the formation of species, identifying the specific genetic basis of either process has been limited to an extremely small number of taxa for which the appropriate genetic tools have been developed. The release of this cricket Gene Index will allow researchers to build on the genetic work of Hoy and Paul [56], which demonstrated a polygenetic basis of cricket songs, and Shaw [58,66], which supported Hoy and Paul's findings and identified several chromosomal regions associated with song, by providing the tools necessary to identify specific genes involved in cricket stridulation, sexual isolation and the formation of species. Identifying the genes involved in any of these processes would represent a significant achievement.

**Figure 1:**
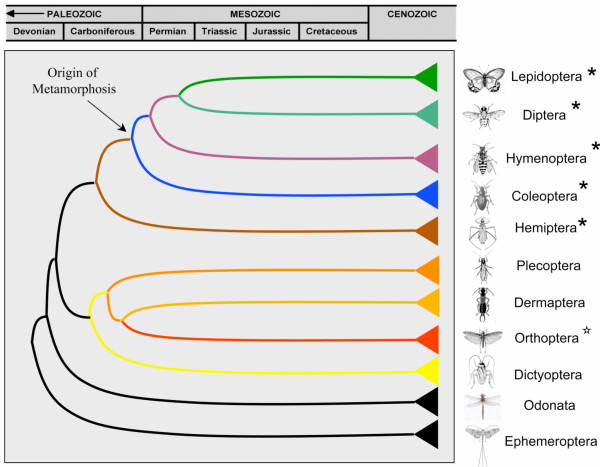
**A Simplified winged-insect phylogeny showing the evolutionary origin of complete metamorphosis (adapted from Grimadi and Engel 2005; Figure 4.24, page 146**.

**Figure 2:**
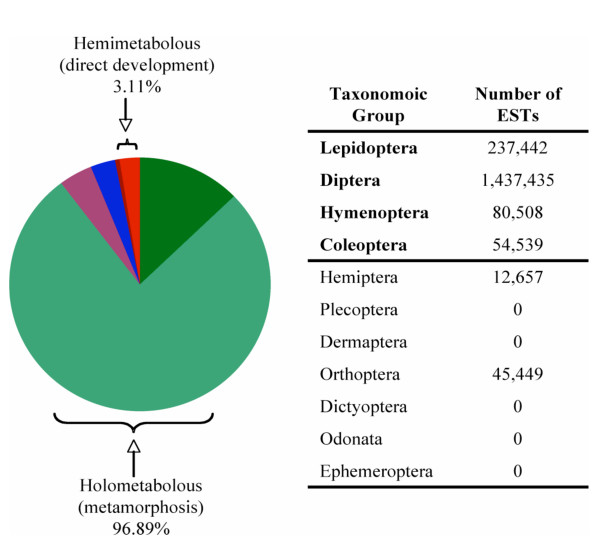
**Pie chart showing the heavy phylogenetic bias towards Holometabolous insects in the total number of EST's deposited in NCBI's dbEST database [105]**.

From a comparative perspective, the publication of the *Laupala *Gene Index is a significant advancement in the tools available to study molecular evolution in insects. To date, major insect genome projects have focused primarily on the Diptera (e.g., fruitflies and mosquitoes; [1,2]), Hymenoptera (e.g. honeybee; [67]), and Lepidoptera (moths and butterflies; [68-70]). All of these lineages belong to a single superorder (Endopterygota) and, thus, represent only a small portion of the phylogenetic diversity encompassed by the broader class Insecta (Figure 1 &2). While the evolution of complete metamorphosis (Holometabolous, Endopterygota) was certainly one of the most significant events in the history of insect diversification [71], the heavy phylogenetic bias of previously developed genomic resources has severely limited broader inferences about the evolutionary history of insects in general. Indeed, only recently have researchers begun to address this phylogenetic bias in studies of arthropod evolution [72,73] and the genomes of an Aphid [74] and Louse [75] soon will be available. Therefore, the compilation of a basal insect genomic resource, such as the one presented here, will facilitate genomic comparisons across 350 million years of insect diversification, and will serve as a phylogenetic link to even more distant comparisons, such as crustaceans (e.g.*Daphnia*) and chelicerates (e.g. tick), and beyond. For example, one of the early developmental studies of arthropod body patterning genes utilized EST sequences cloned from *Schistocerca *(Orthoptera: Caelifera) and *Tribolium *(Coleotpera) to demonstrate the homology between the *Drosophila *hox gene *zen *and its' human ortholog, HOX3 [76]. Thus, the benefits of developing sophisticated genomic resources for non-model organisms are potentially much broader than typically recognized.

The current study represents the first major initiative to develop a large genomic resource for a cricket species of the orthopteran suborder Ensifera (crickets and katydids). We present the sequences of 14,502 Expressed Sequence Tags (EST) from a *Laupala kohalensis *nerve cord cDNA library. We expect that the release of this Gene Index will provide much needed tools for the study of CPG construction and evolution, sexual selection and speciation, and the molecular evolution of arthropods.

## Results

Two separate, normalized cDNA libraries were constructed from a single pool of RNA extracted from the nerve cord tissue of several individual crickets. A total of approximately 22,000 clones were isolated from these libraries. 388 clones were sequenced from the first library (LK01); 14114 clones were sequenced from the second library (LK04). A total of 14,502 sequences were generated. Preliminary sequence analysis revealed that 5' end sequencing of the EST's provided higher quality reads than those generated from the 3' end. As a result, the majority of our sequencing effort was directed at sequencing the 5' end of the EST's. 14,261 sequences were generated from the 5' end and 241 sequences were generated from the 3' end of the insert. Of the 14,502 sequences, 14,377 were greater than 100 bases after the vector and linker sequences were stripped. Of these 14,377 sequences, read lengths ranged from 100 bases to 1051 bases. The average read length was 704 bases. Table 1 summarizes the results of the cDNA sequencing and basic bioinformatics analysis. All 14,377 sequences were submitted to GenBank and can be accessed through the accession numbers EH628894-EH643270.

A Gene Index was created from these 14,377 acceptable sequences [77]. We identified 8,607 unique sequences, representing 6,032 singletons and 2575 tentative consensus sequences (TCs). Tentative consensus sequences are composed of multiple sequencing reads with overlapping sequence alignments. The 2,575 TCs were derived from 8,345 EST's (Table 2) and ranged in length from 167 bases to 3,317 bases, with an average length of 935 bases. The number of EST's per TC ranged from 2 to 41, with a mean number of 3.24 EST's per TC. The remaining unique sequences were composed of single EST's. Singleton sequences ranged in size from 102 bases to 1019 bases, with an average length of 700 bases (Table 3).

The 8,607 unique sequences were translated into all 6 possible reading frames and compared using BLAT [78] against a comprehensive non-redundant protein database maintained by the Dana-Farber Cancer Institute. This database contains ~3 million entries collected from UniProt, SwissPro, RefSeq, GenBank resources and additional sequences from TIGR and its affiliates. The BLAT algorithm is integrated into the gene indexing bioinformatics pipeline to reduce computing times when building and annotating other large gene indices (e.g. human, [79]; mouse, [80]; and rat, [81]). In future releases, the pipeline may be modified to use additional algorithms, such as BLASTX, when working with more limited and/or phylogenetically distinct gene indices such as our cricket gene index.

5,225 of the 8,607 (60.7%) unique sequences had a significant sequence similarity match to an entry in the protein database [see Additional file 1]. 3,382 (39.3%) unique sequences returned no significant matches to entries in the database and no putative function could be assigned to them. However, 2,393 of the 3,382 (70%) sequences that did not return a significant match to a protein in the database were identified by ESTscan [82] as having putative ORF's with an average length of 295 nucleotides. This suggests that the majority of these unidentified EST's are expected to encode a protein and highlights the dearth of genomic information available for basal insect taxa.

The observed sequence similarities produced by the comparative analysis are consistent with our expectations given the tissue from which the cDNA library was constructed. While some of the unique sequences are similar to housekeeping genes, many unique sequences are similar to genes that may influence stridulation (Table 4). For example, several unique sequences are similar to genes that regulate the timing of biological events (e.g. Period and Diapause bioclock protein; Table 4), while others are involved with nervous system signal transduction (e.g. cGMP-gated cation channel protein, G-protein-coupled receptor, Shab-related delayed rectifier K+ channel, Na+/K+/2Cl-cotransporter, Nicotinic acetylcholine receptor non-alpha subunit precursor, Potassium channel tetramerisation domain-containing protein 5, Voltage-dependent anion channel, and Syntaxin 7; Table 4) and others contribute to developmental events that shape either the nervous system (e.g. Even-Skipped; Table 4) or wing development (e.g. Notch, Wnt inhibitory factor 1; Table 4). In addition to potentially influencing our primary phenotype, many of these sequences will be useful to researchers interested in insect neural function (e.g. Calmodulin, Innexin; Table 4) and insect molecular evolution (e.g. Opsin, Dyenin; Table 5).

Within our unigene set, we identified a number of genes that would be of comparative interest. To explore the *Laupala *unigene set as a comparative utility we compared the sequence of ten EST's from our unigene set to unigene sets available in *Drosophila melanogaster*, *Anophelese gambiae*, *Bombyx mori*, *Apis mellifera*, *Tribolium casteneum*, and *Locusta migatoria *(Table 5). The results show the evolutionary distinctiveness and phylogenetic distance between *Laupala *sequences and EST sequences from other genomic models. Across the ten EST's, the mean uncorrected sequence divergence (*p*) between *Laupala *and the other insect taxa surveyed was 30%. Furthermore, the mean distance between *Laupala *and *Locusta *was 89% that of the mean pairwise distance of all taxa in the analysis. Thus, despite the fact that *Laupala *and *Locusta *are both members of the insect order Orthoptera, the sequence divergence between them for this sample of EST's is close to that found among other insect orders.

**Figure 3:**
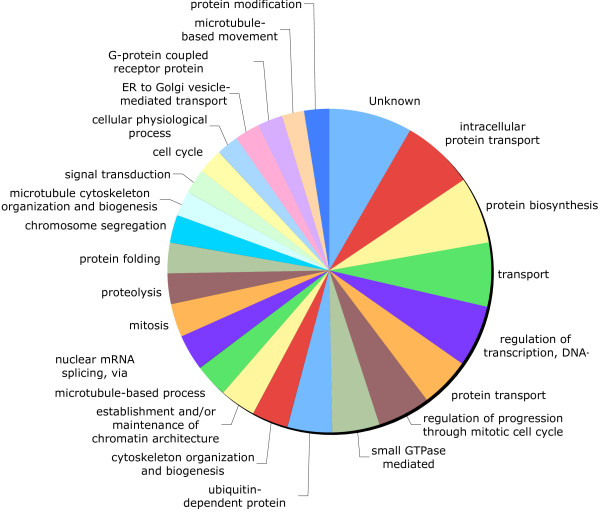
**A piechart of the 25 most frequent Biological Process Gene Ontology (GO)terms**.

**Figure 4:**
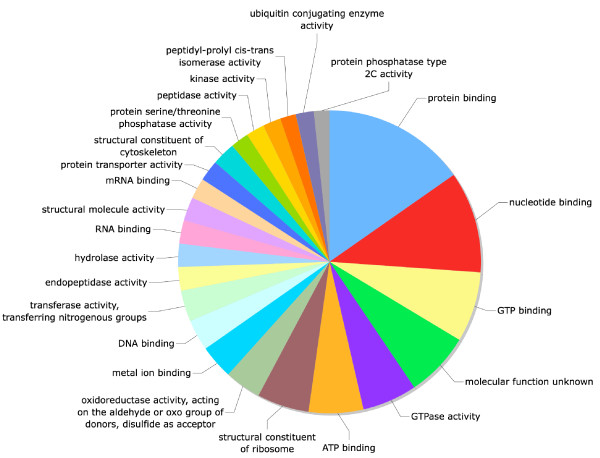
**A piechart of the 25 most frequent Molecular Function Gene Ontology (GO)terms**.

**Figure 5:**
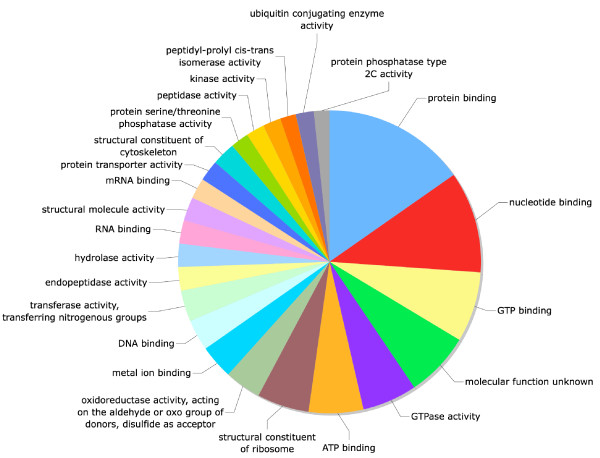
**A piechart of the 25 most frequent Cellular Compartment Gene Ontology (GO)terms**.

Of the 5,225 sequences that matched protein entries, 408 sequences could be assigned a Gene Ontology (GO, [83,84]) term (Figures 3,4,5). 572 Biological Process GO terms were associated with predicted amino acid sequences from these 408 sequences. The 25 most frequent Biological Process GO terms are presented in Figure 3. The majority of Biological Process GO terms (488 or 85%) were assigned to five or fewer of the 408 sequences present and no Biological Process GO term was assigned to more than 45 sequences. 275 Molecular Function GO terms were associated with amino acid sequences identified in the 408 unique sequences. The 25 most frequent Molecular Function GO terms are presented in Figure 4. The majority of Molecular Function GO terms (221 or 80%) were assigned to five or fewer sequences. One Molecular Function GO term was assigned to 100 of the 408 sequences (protein binding). 212 Cellular Compartment GO terms were associated with predicted amino acid sequences identified in the 408 unique sequences. The 25 most frequent Cellular Compartment GO terms are presented in Figure 5. The 408 unique sequences contained 106 predicted nuclear proteins, and this was the most frequent Cellular Compartment GO term. Again, the majority of these GO terms, 163 (77%), were assigned to no more than five of the 408 sequences.

The low redundancy of the GO terms, in addition to the large proportion of singletons in the library and the small number of EST's per TC, testify that the normalization was successful and that a large proportion of the genes expressed in the cricket developing nerve cord were identified. The putative function of the singletons and tentative consensus sequences, as inferred from the BLAT comparison and the GO term assignments, is consistent with genes expected to be expressed in a nerve cord.

## Discussion

We completed an EST sequencing project to characterize genes expressed in the cricket nerve cord that underlie pulse rate of male song in *L. kohalensis*. By constructing a cDNA library from nymphal and adult crickets, our aim was to enhance the discovery of genes involved in the construction of the central pattern generating circuit (CPG) underlying rhythmic singing behavior. In addition, we enriched for full-length cDNA by utilizing a template-switching reverse transcriptase (SMART™ technology – BD Clontech, Mountain View, CA). Furthermore, we increased the representation of genes expressed in low-copy number by normalizing our amplified cDNA using a double-stranded nuclease (Trimmer-Direct Kit; Evrogen, Moscow). Sequencing of ~22,000 clones from this library by The Institute for Genomic Research (TIGR) produced 14,502 high quality EST's with an average length greater than 700 bases (Tables 1, 2, 3). Assembly of these EST's produced 8,607 unique sequences. We were then able to annotate 5,225 of these genes based on BLAT protein comparisons against a comprehensive non-redundant protein database maintained by the Dana-Farber Cancer Institute. Of these annotated genes, we could assign gene ontology (GO) terms to 408 genes. The diversity of our library is reflected in the large number of different GO terms assigned to these genes, including 572 Biological Process, 275 Molecular Functions, and 212 Cellular Compartment GO terms, and suggests that we were successful in our attempt to normalize cDNA representation in our library.

### Cricket Gene Index

A Gene Index based on our EST sequencing project was assembled and is publicly-available at [85]. This electronic resource consists of a description of the cricket EST library, including a summary of the number of unique sequences, the distribution of tentative consensus (TC) sequences, gene annotations, GO terms, and a set of 70-mer oligonucleotide probes. The cricket Gene Index thus joins more than 30 other animal gene indices hosted by DFCI and represents the second largest EST resource for Orthoptera available online. While the cricket EST project sequenced roughly one third of that sequenced by the *Locusta migratoria *project (45,754 EST's, [86]) this disparity is not reflected in the total number of unique sequences identified by these two projects (*L. migratoria *= 12,161 unique sequences versus *L. kohalensis *= 8,607 unique sequences).

### Crickets as models for behavioral genomics

Species of Orthoptera have long served as neurophysiological models of behavior. Our analysis of 14,502 EST sequences and subsequent production of 8607 singletons and tentative consensus sequences from a nerve cord derived library represents a major advance in the available genomic resources for the study of cricket neurophysiology and behavior. This resource will provide valuable tools with which to examine the underlying genetic basis of cricket stridulation, a model for the study of central pattern generation (Table 4). The resources presented here represent the first opportunity to analyze the neurophysiologic process of stridulation at the genomic scale.

### Developing additional genomic resources for *Laupala*

We are utilizing multiple approaches in order to dissect the genetic basis of pulse rate variation in *Laupala*. In addition to ongoing QTL mapping efforts [64] (Shaw et al. in press), the *Laupala *Gene Index is a first step towards two additional genetic approaches to our study of pulse rate evolution. First, the oligonucleotide probe set developed from our Gene Index is the backbone of an oligonuclelotide micoarray being constructed to study gene expression in *Laupala*. These microarrays will be used to study patterns of gene expression across multiple species [87] to identify candidate genes whose expression varies with pulse rate. Second, the EST's are being screened for variation that can be used in a linkage analysis. Placing these EST's on the *Laupala *linkage map will facilitate comparisons between the QTL analysis and the study of gene expression. The identification of candidate genes that fall within QTL regions will strengthen the support for these candidate genes and guide our choice of which genes to use in functional studies. Furthermore, estimating the linkage relationships of EST's within *Laupala *and comparing them with known orthologs in model systems will allow us to identify regions of synteny across multiple species. Establishing such areas of synteny is another powerful approach to identifying strong candidate genes [88-90]. Given the now rich genomic resources available in *Laupala*, the extensive divergence of male song CPG and its influence on reproductive isolation, and the fairly limited genetic divergence within this genus, *Laupala *represents an excellent system to study the evolutionary genomics of CPG diversification.

In addition, the development of genomic resources in *Laupala *can be used to tackle some of the most urgent topics in evolutionary biology. Few other systems provide both the genomic tools and evolutionary power necessary to provide an understanding of how gene expression evolves in recently diverged taxa [91]. Furthermore, because male pulse rate plays a critical function in reproductive isolation in this genus, identifying the genes whose expression contributes to the construction of this phenotype will provide insight into how the evolution of gene expression contributes to reproductive isolation during the course of speciation [92].

### Comparative genomics in insects

In the last 15 years, there has been a proliferation of genomic resources available for model organisms. As technology has improved, whole genome sequences have become available for a growing number of species and for the first time comparative studies of entire genomes have become possible [93-96]. However, the phylogenetic breadth of insect species in which genomic tools have been developed is extremely limited. For example, of the 37 insect genomes sequencing projects currently completed or under way, 22 (~60%) involve species of *Drosophila*. The remaining species are either directly related to human health (the mosquitoes *Aedes aegypti *and *Culex pipiens*, the Tsetse fly *Glossina morsitans*, the human louse *Pediculus humanus humanus*, and the Hemipteran vector of Chaga's disease *Rhodnius prolixus*) [97], or are of agriculture importance (the red flour beetle *Tribolium casteneum*, the honey bee *Apis mellifera*, the silkworm moth *Bombyx mori*, the pea aphid *Acyrthosiphon pisum*, and the parasitoid wasp *Nasonia vitripennis*). The only species with significant genomic tools that is not of biomedical or agricultural importance is the African butterfly (*Bicyclus anyana*), an evo-devo model for wing pattern development [98]. The vast majority of these insects are holometabolous and possess relatively small genomes [99,100]. However, this severe phylogenetic and genome-size bias limits comparative studies of insect and arthropod evolution (Figure 1 &2). The cricket Gene Index presented here represents a significant contribution to the genomic resources available for comparative molecular studies of basal insect lineages (Table 5). Based on our preliminary comparative analysis, *Laupala*, a representative of the Orthopteran suborder Ensifera, is as distinct from *Locusta*, a representative of the Califeran suborder of the Orthoptera, as it is from other insect orders.

## Conclusion

We document the sequencing of 14,502 EST's derived from a *Laupala kohalensis *nerve cord cDNA library. From these 14,502 sequences, 8,607 unique sequences were identified. Just over 60% of the unique sequences, 5,225, had a predicted protein sequence significantly similar to a sequence in a non-redundant protein database. Of these, Gene Ontology terms could be assigned to 408 of the putative proteins. This resource was developed to address fundamental questions of biological interest. Our interests lie in identifying genes that contribute to the diversification of male song pulse rate and, by extension, speciation within the Hawaiian cricket genus *Laupala*. The release of this resource, however, has a much broader impact than that prescribed by our interests. Neuroethologists studying the construction and function of CPG neural circuits in insects have lamented the lack of available genetic tools necessary to study these vital neurobiological phenotypes. The release of the *Laupala *Gene Index contributes to meeting this need. Likewise, evolutionary biologists have lacked diverse systems with which fundamental evolutionary processes might be addressed at the genomic scale. Empirical data can be collected using the *Laupala *resource to examine the evolution of gene expression during the speciation process. Finally, the release of this Gene Index begins to rectify an extreme phylogenetic bias in the availability of genomic resources in insects and will facilitate comparative studies of molecular evolution across 350 MY of arthropod evolution.

## Methods

### Cricket rearing and RNA isolation

*Laupala kohalensis *were raised from laboratory-reared parents under identical and constant light (12:12) and temperature (20°C) conditions. Crickets were fed Cricket Chow (Purina) twice weekly. Groups of crickets were reared in quart-sized, glass jars outfitted with moistened Kimwipes (Kimberly-Clark) from hatching. As individuals matured to approximately the 5^th ^post-embryonic instar, 2–4 individuals per group were moved into individual specimen cups and maintained under conditions identical to the jars.

Between the hours of 08:00 and 12:00, groups of crickets were anaesthetized with carbon dioxide, and individuals were digitally imaged using a Leica MZ8 compound microscope mounted with a JVC TK-1280U camera connected to a Power Macintosh 7500/100 Apple computer via the program NIH Image. Individuals were transferred to Corning 1 ml cryovials and snap frozen through the emersion of the cryovials into liquid nitrogen and immediately moved to -70°C. All crickets were sacrificed at 12:00.

The individuals included in this study spanned the putative critical developmental period (instars 5–8) during which the neural circuit responsible for orthopteran stridulation is established [2]. 17 crickets were individually thawed under RNAlater (Ambion) and dissected to remove the nerve cord. Based on the width of the pronotum, individuals were assigned to one of 8 post-embryonic developmental stages [27]. Of the 17, 8 and 6 were sacrificed at instars 5 and 6, respectively. At these stages, neither wing buds nor ovipositors are apparent; therefore the gender could not be determined for these individuals. In addition, two males at instar 7, and one female at instar 8 were included in the study.

RNA was extracted from the pooled, dissected nerve cord using an RNAeasy mini (Qiagen) kit in combination with a QiaShredder column (Qiagen). The quality and quantity of RNA was assessed via spectrometry at 260 nm and 280 nm.

### cDNA synthesis

Double-stranded cDNA was synthesized from total RNA isolated from nerve cord tissue of *L. kohalensis *using the Creator™ SMART™ system developed by Clontech BD Bioscience (Mountain View, CA). This method combines long-distance PCR with a proofreading polymerase and a template switching reverse transcriptase to preferentially amplify full-length cDNA's. During the first-strand synthesis, short universal priming sites with asymmetrical *Sfi*I digestion sites are incorporated to both the 5' and 3' ends of each cDNA fragment. A second round of amplification is then performed via primer extension [101] to generate double-stranded cDNA that can then be digested and directionally cloned into an appropriate vector.

Reaction conditions for the first-strand synthesis were as follows: 2 μl of total RNA from either *Laupala *nerve cord tissue (~0.8 μg/μl) or control Human placenta (1.0 μg/μl), 1 μl of RNAse-free water (Ambion), 1 μl of the 5' SMART IV™ primer (BD Clontech), and 1 μl of a 3'oligo d(T) primer with a modified adaptor (CDS-3M – Evrogen, Moscow) were incubated at 72°C for 2 minutes and then placed on ice for an additional 2 minutes. To this reaction, 2 μl of 5× 1^st ^strand buffer, 1 μl of DTT (20 mM), 1 μl dNTPs (10 mM), and 1 μl of PowerScript™ reverse transcriptase were added and the mixture was incubated at 42°C for 90 minutes. 2 μl of the first-strand template was used in the second-strand reaction in 100 μl total volume under the following cycling conditions: an initial 95°C incubation for 1 minute, 16 cycles of (95°C for 30 s, 66°C for 30 s, and 72°C for 4 minutes), and a final 72°C incubation. 5 μl of this PCR product were then visualized on a 1.0% agarose gel to assess the quality of the amplification.

### cDNA normalization

We normalized our library using a Trimmer-Direct cDNA normalization kit (Evrogen, Moscow) to reduce the abundance of high copy number cDNA and to increase the probability of cloning and sequencing low copy number cDNA's. Briefly, purified cDNA (~1000 ng) was denatured at 95°C and then incubated at 68°C in hybridization buffer for 5 hours. Following this incubation, cDNA was exposed to a double-stranded nuclease enzyme (DSN, Evrogen) at three different concentrations (1,1/2, and 1/4) for 25 minutes at 68°C. This reaction was stopped by a 5 minute incubation on ice. The normalized cDNA was then amplified using primers complementary to the adaptors incorporated during the second-strand reaction. Initial amplification consisted of 7 cycles of 95°C for 30 s, 66°C for 30 s, and 72°C for 4 minutes. The reactions were the placed at 4°C while non-normalized controls were cycled for an additional 6 cycles. Aliquots of these controls were removed at 9, 11, and 13 cycles. These products were visualized to determine the optimal number of cycles, and based on these results the normalized cDNA amplifications were placed back in the theromcycler for an additional 13 cycles (total # of cycles = 20).

5 μl aliquots of the amplified, normalized cDNA from each of the 3 different DSN enzyme treatments were run out on an agarose gel along side un-normalized control (Human placenta) and experimental (*Laupala *nerve cord) cDNA PCR products. Visualization indicated that the 1/2 DSN and 1/4 DSN enzyme concentrations both normalized the cDNA well. Treatment with the full strength enzyme had over-degraded the samples. Therefore, we combined the normalized cDNA PCR products for the two diluted DSN treatments. This template was then used for a final round of amplification (12 cycles: 95°C, 64°C, and 72°C for 30 s) before cloning the normalized cDNA into pDNR-lib vector (BD Clontech).

### Size-fractionation, directional cloning, and transformation of normalized cDNA

The amplified cDNA was digested with *Sfi*I (79 μl of normalized cDNA, 10 μl of NEB buffer 2, 10 μl restriction enzyme, and 1 μl ob BSA) for 2 hours at 50°C, and then the cDNA was ethanol precipitated and resuspended in 10 μl of RNAse-free water. *Sfi*I digestion results in asymmetrical sticky-ends on all of the cDNA fragments and permits directional cloning. We combined several separate digestion aliquots to concentrate the cDNA. Cleaned, digested fragments were allowed to run out on a 1% agarose gel for 6 hours at low voltage to ensure good size separation. We size-fractionated the library to enrich for fragments between 1.5 kb and 4 kb. The cDNA was gel-purified and resuspended in RNAse-free water. We ligated the normalized cDNA into pDNR-lib, a plasmid vector specifically designed for cDNA library construction, and incubated these reactions at 16°C overnight. The ligations were ethanol-precipitated and resuspended in 10 μl of RNAse-free water. 2 μl (~800 ng) of the ligated vector was used to transform electro-competent cells (ElectroTen-Blue. Stratagene, La Jolla, CA) which were then grown for an hour in LB media. A serial titration was used to titer the library and to determine the number of positive transformants. Average insert size was estimated by amplifying 96 randomly chosen clones.

### EST sequencing

Each library was spread on LB-Agar plates containing 100 ug/ml of chloramphenicol. Positive transformants were identified and isolated using a Q-Pix automated colony picker. Isolated clones were grown overnight in LB at 37° at 900 RPM. Plasmid DNA was isolated using a modified alkali lysis method and was used as a template in a sequencing reaction. Either M13 forward or M13 reverse was used to prime the sequencing reaction. Randomly selected clones from the two libraries were sequenced using dye-terminator chemistry (Applied Biosystems) with ABI 3730 automated sequencers. Individual nucleotides were called using TraceTuner 2.0 (Paracel), and sequence reads with quality score >20 were used to construct a cricket Gene Index.

### Cricket Gene Index assembly and annotation

The cricket Gene Index database was assembled at Dana-Farber Cancer Institute as described elsewhere [102]. Cricket EST reads of sufficient quality were first subjected to a vigorous screening procedure to identify and remove the contaminating vector and adaptor sequences, poly-A/T tails, and bacterial sequences. EST's shorter than 100 bases after trimming were discarded, and the remaining 14,377 cleaned sequences were compared pair-wise using a modified version of the MegaBLAST program [103] that eliminates the generation of the final alignment lay-out to speed up the process. Following this initial pair-wise search, sequences sharing greater than 95% identity over at least 40 bases and with less than 20 bases unmatched sequence at either end were grouped into clusters, leaving unclustered sequences as singletons. Components of each cluster were then assembled using the Paracel Transcript Assembler (PTA), a modified version of CAP3 assembly program [104] to produce Tentative Consensus (TC) sequences. These virtual cDNA's with assigned TC numbers together comprise the cricket Gene Index. Following assembly, TCs and singleton EST's were searched against a non-redundant protein database using the BLAT program [78], and assigned a provisional function if they had hits exceeding a threshold BLAT score of 30 and a 30% similarity cutoff. cDNA's with high-scoring hits were also annotated with Gene Ontology (GO) terms and Enzyme Commission (EC) numbers and Kyoto Encyclopedia of Genes and Genomes (KEGG) metabolic pathway information using a SwissProt to GO translation table provided by the GO consortium.

### Comparative analysis

To demonstrate the phylogenetic distinctiveness of these data, ten *L. kohalensis *unigenes were chosen based on their annotation results for a comparative analysis of sequence evolution. These 10 unigenes were translated in all 6 possible reading frames and compared using BLAT to a database containing the 6 possible reading frame translations of the unigene sets from the following organisms: *Drosophila melanogaster*, *Anophelese gambiae*,*Bombyx mori*, *Apis mellifera*, *Tribolium casteneum*, and *Locusta migratoria*. The unigene with the highest BLAT score from each of the species in the database, when one could be identified, was selected.

EST's that returned a significant BLAT hit to the *Laupala *sequences were aligned using a weighted CLUSTAL algorithm and default alignment parameters in the program MegAlign (DNASTAR, Inc, Madison, WI). Aligned datasets were then exported as NEXUS files [see Additional file 2, see Additional file 3, see Additional file 4, see Additional file 5, see Additional file 6, see Additional file 7, see Additional file 8, see Additional file 9, see Additional file 10, see Additional file 11, see Additional file 12] and analyzed further in PAUP * 4.0b10 (Swofford 2000). Uncorrected distances (p-distances) were calculated for all pairwise comparisons. Gene regions compared included only those with representation from all organisms; other regions were excluded from analyses. Regions with substantial gaps in alignment were also excluded.

## Authors' contributions

PDD participated in the conception of the project, the design of the study, the creation of the cDNA library and the drafting of the manuscript. SPM participated the design of the study, the creation of the cDNA library and the drafting of the manuscript. JQ and FL participated in the construction of the cricket Gene Index from EST sequences and making the resources accessible online. VN participated in establishing the collaboration and DNA sequencing. KLS participated in the conception of the project, the design of the study, establishing the collaboration and the drafting of the manuscript. All authors read and approved the final manuscript.

## Supplementary Material

Additional file 1 BLAT best hits results. This is a text file that lists the top BLAT matches for each of the 5,225 unique sequences with significant sequence similarity to known proteins. Click here for file

Additional file 2 Characters used in the gene alignments for comparative analysis. This file identifies the characters used in the comparative analysis of the 10 unigenes presented in Table 5. Click here for file

Additional file 3 NEXUS file of Actin alignment. This file presents the alignment of the six actin sequences used for comparative analysis. Click here for file

Additional file 4 NEXUS file of alpha-tubulin alignment. This file presents the alignment of the six alpha-tubulin sequences used for comparative analysis. Click here for file

Additional file 5 NEXUS file of alpha-tubulin alignment. This file presents the alignment of the six alpha-tubulin sequences used for comparative analysis. Click here for file

Additional file 6 NEXUS file of dynein (light chain) alignment. This file presents the alignment of the six dynein (light chain) sequences used for comparative analysis. Click here for file

Additional file 7 NEXUS file of histone 2a alignment. This file presents the alignment of the six histone 2a sequences used for comparative analysis. Click here for file

Additional file 8 NEXUS file of HSP40 alignment. This file presents the alignment of the six HSP40 sequences used for comparative analysis. Click here for file

Additional file 9 NEXUS file of malate esterase alignment. This file presents the alignment of the six malate esterase sequences used for comparative analysis. Click here for file

Additional file 10 NEXUS file of myosin 2 (light chain) alignment. This file presents the alignment of the six myosin 2 (light chain) sequences used for comparative analysis. Click here for file

Additional file 11 NEXUS file of opsin alignment. This file presents the alignment of the six opsin sequences used for comparative analysis. Click here for file

Additional file 12 NEXUS file of polyubiquitin alignment. This file presents the alignment of the six polyubiquitin sequences used for comparative analysis. Click here for file

## Figures and Tables

**Table 1:** Sequencing results of the two libraries which were examined including raw sequencing results and acceptable sequences after removing poor quality reads and contaminating sequences.

	**Pooled LK libraries**			**Library LK01**			**Library LK04**		
**EST Sequence Total Reads**	**all reads**	**5' end reads only**	**3' end reads**	**all reads**	**5' end reads only**	**3' end reads**	**all reads**	**5' end reads only**	**3' end reads**
			
Number of Successful Sequences	14502	14261	241	388	316	72	14114	13945	169
Range in Length	241–1252	268–1252	241–1128	758–1150	958–1150	758–1102	241–1252	268–1252	241–1128
Mean Length	1057	1058	1024	1082	1092	1041	1057	1057	1017
**High Quality EST Reads**									
Number of Successful Sequences	14502	14261	241	388	316	72	14114	13945	169
Range in Length	64–1096	64–1096	66–1051	68–1074	218–1074	68–943	64–1096	64–1096	66–1051
Mean Length	838	841	619	805	875	499	838	840	670
**EST Sequence After Vector Stripping**									
Number of Successful Sequences	14377	14158	219	354	295	59	14023	13863	160
Range in Length	100–1051	100–949	103–1051	100–926	100–926	105–916	100–1051	100–949	103–1051
Mean Length	704	705	657	486	473	553	710	710	695

**Table 2:** Statistics of Tentative Consensus sequences (TCs)

Number of TC	2575
Number of ESTs assembled into TC	8345
TC size range (bp)	167–3317
Mean TC length (bp)	935
Range of number of EST's in TC	2–41
Average number of EST's in TC	3.24
Number of TC with >= 20 EST's	17
Number of TC with < 5 EST's	2205

**Table 3:** Statistics of singletons

Number of singletons	6032
Singleton size range (bp)	102–1019
Mean singleton length (bp)	700
Number of singletons <= 200 bp	110
Number of singletons between 200 and 500 bp	505
Number of singletons between 500 and 800 bp	3860
Number of singletons > 800 bp	1557

**Table 4:** Genes of neurobiological interest

Sequence ID	Gene
TC1375	Calmodulin
1099956307901	Calpain B
1099956293105	cAMP-dependent protein kinase subunit R2 beta
1099956429052	cGMP-dependent protein kinase
TC588	cGMP-gated cation channel protein
TC140	Diapause bioclock protein
TC1309	Even-Skipped
1099956350726	G-protein-coupled receptor
1099817827099	Innexin
1099817862791	Intersectin-1
TC1333	Membrane-associated ring finger
1099956579253	MscS Mechanosensitive ion channel
1099956736101	Myosin V
1099956378602	Na+/K+/2Cl-cotransporter
TC1855	Nicotinic acetylcholine receptor non-alpha subunit precursor
TC2167	Notch
1099956498166	Period
TC1283	Potassium channel tetramerisation domain-containing protein 5
1099956317550	Rab7
TC1866	Ras-related protein Rab-2
1099956329054	Serpentine Receptor
TC1295	Shab-related delayed-rectifier K+ channel
1099956378537	sodium and chloride-dependent high-affinity choline transporter
TC456	Sparc
TC2021	Stathmin
1099817880653	Swelling dependent chloride channel
1099817832930	Syntaxin 7
1099956598763	Troponin T
TC2416	Voltage-dependent anion channel
1099956851891	Wnt inhibitory factor 1

**Table 5:** Genes of comparative interest. Uncorrected distances between *Laupala *and the specified taxon are shown, where possible. The mean uncorrected pairwise distance (*p*) between all taxa (excluding *Laupala*) is shown for each gene in the final column for comparison. Alignments of each gene are presented as NEXUS files in the online additional files.

	***Locusta***	***Tribolium***	***Apis***	***Bombyx***	***Anopheles***	***Drosophila***	**Mean Distance (excluding *Laupala*)**
**Actin**	0.0911	0.1752	0.1262	0.1594	0.1051	0.0911	0.1368
**Alpha-tubulin**	0.2090	0.2143	0.2288	0.1744	0.2135	0.1878	0.2115
**Aquaporin**	0.3164	0.4715	0.4242	0.4814	0.4400	0.4336	0.4485
**Dynein (Light Chain)**	0.1741	0.2482	0.1741	0.6043	0.2185	0.2037	0.2111
**Histone 2A**	0.3184	0.2720	0.3081	0.2478	0.2016	0.3218	0.3039
**HSP40**	0.3959	0.4832	0.3592	0.3392	0.3587	0.4049	0.4287
**Malate Esterase**	0.3056	0.4032	0.3526	-	0.4140	0.4430	0.3802
**Myosin 2 (Light Chain)**	0.2576	0.3529	0.3132	0.3352	0.4254	0.3856	0.3652
**Opsin**	0.3430	-	0.3630	-	0.4173	0.4387	0.3911
**Polyubiquitin**	0.2046	0.2292	0.2237	0.2046	0.2846	0.2194	0.2321
