# Supplementary material for: A cricket Gene Index: a genomic resource for studying neurobiology, speciation, and molecular evolution
Source: BMC Genomics. 2007 Apr 25;8:109. doi: 10.1186/1471-2164-8-109 (PMC1878485; doi:10.1186/1471-2164-8-109)
Supplement: Additional file 2 — Characters used in the gene alignments for comparative analysis. This file identifies the characters used in the comparative analysis of the 10 unigenes presented in Table 5. [file 1471-2164-8-109-S2.doc]

Actin

Characters used for p distance: 984-1411

Alpha tubulin

Characters used for p distance: 226-982

Aquaporin

Characters used for p distance: 290-944

Dynein Light Chain

Characters used for p distance: 208-482

Histone 2A

Characters used for p distance: 56-505

HSP40

Characters used for p distance: 423-1011

Malate Esterase

Characters used for p distance: 150-837

Myosin_2 Light Chain

Characters used for p distance: 614-1085

Opsin

Characters used for p distance: 548-1198

Polyubiquitin

Characters used for p distance: 430-2052* for comparisons EXCLUDING Anopheles

Characters used for p distance: 430-1478* for comparisons WITH Anopheles
